# Investigation of the distribution and origin of porcine reproductive and respiratory syndrome virus 1 in the swine production chain: A retrospective study of three farms in Thailand

**DOI:** 10.14202/vetworld.2024.1722-1732

**Published:** 2024-08-04

**Authors:** Tippawan Jantafong, Wimontiane Saenglub, Nattarun Chaisilp, Weena Paungpin, Thatsanee Tibkwang, Pattama Mutthi, Teerawin Bouma, Porntippa Lekcharoensuk

**Affiliations:** 1 Department of Preclinical Sciences, Faculty of Veterinary Medicine, Mahanakorn University of Technology, Bangkok 10530, Thailand; 2 Department of Microbiology and Immunology, Faculty of Veterinary Medicine, Kasetsart University, 50th Ngamwongwan Rd., Chatuchak, Bangkok, 10900, Thailand; 3 The Monitoring and Surveillance Center for Zoonotic Diseases in Wildlife and Exotic Animals, Faculty of Veterinary Science, Mahidol University, Nakhon Pathom 73170, Thailand; 4 Office of the Dean, Faculty of Veterinary Science, Mahidol University, Nakhon Pathom 73170, Thailand; 5 Faculty of Veterinary Medicine, Rajamangala University of Technology Tawan-ok, Chonburi 20110, Thailand; 6 Animal Production Innovation and Management Division, Faculty of Natural Resources, Prince of Songkla University, Hat Yai Campus, Songkhla, 90110, Thailand

**Keywords:** porcine reproductive and respiratory syndrome virus-1, swine production system, Thailand, virus distribution

## Abstract

**Background and Aim:**

Porcine reproductive and respiratory syndrome (PRRS), caused by PRRS virus (PRRSV), is a global issue that affects Thai swine as well. In Thailand, PRRSV-2 predominates over PRRSV-1. The origin of PRRSV-1 transmission remains undiscovered. This study traced the source of infected pigs responsible for disease transmission among three pig-fattening farms and analyzed the spread of PRRSV-1.

**Materials and Methods:**

A total of 696 swine samples from breeding and pig-fattening farms in Thailand were screened for PRRSV using open reading frames (ORF7) reverse transcription polymerase chain reaction (RT-PCR). Positive samples were identified as PRRSV-1 using ORF5 RT-PCR. The analysis included the study of nucleotide homology, GP5 amino acid sequences, and N-linked glycosylation patterns to assess the spread of PRRSV-1 across these farms.

**Results:**

Genetic examination identified 28 PRRSV-1-positive samples, of which 13 were chosen as representatives. These strains were categorized into three groups based on breeding farm pig houses and showed distinct distribution patterns across pig-fattening farms. Group 1 included piglets transferred from pig house A to Nakhon Pathom, Chonburi, and Sa Kaeo. Groups 2 and 3 showed transfers from pig houses F and H to Chonburi and Sa Kaeo farms. All 13 PRRSV-1 strains were categorized into PRRSV-1 subtype 1/clade H. N-linked glycosylation analysis revealed that nearly all PRRSV-1 strains exhibited a conserved glycosylation pattern at amino acid positions N37, N46, and N53. This pattern is consistent with the glycosylation profile of the previous Thai PRRSV-1 subtype 1/clade H.

**Conclusion:**

The present study highlights the persistent presence of PRRSV-1 in Thai swine, which leads to sporadic outbreaks. The molecular genetic analysis identified three primary strain groups dispersed throughout the pig production system, emphasizing the importance of regular monitoring for new PRRSV strains in this herd. Understanding the PRRSV-1 distribution in swine farms is vital for veterinarians. This knowledge supports strategies for eradicating the virus and managing swine health effectively in Thailand.

## Introduction

Porcine reproductive and respiratory syndrome (PRRS) is caused by the PRRS virus (PRRSV). Its symptoms include reproductive failure in sows, late-term abortions, and respiratory issues in piglets [1]. In Asia, countries including China, India, Indonesia, Japan, Taiwan, and Thailand have been significantly affected by PRRS, a major transboundary and endemic disease [2–9]. PRRSV can be transmitted through the excreta and secretions of infected pigs, with the potential for airborne transmission [2]. PRRSV can persist in pig herds for extended periods, leading to substantial economic losses [10]. PRRSV is a small, enveloped virus with a positive-sense, single-stranded RNA genome of approximately 15 kb in length [11]. The viral genome comprises 11 open reading frames (ORFs), specifically ORF1a, ORF(TF), ORF1b, ORF2a, ORF2b, ORF3, ORF4, ORF5a, ORF5, ORF6, and ORF7 [12, 13]. PRRSV is classified under the genus *Porartevirus*, within the family *Arteriviridae*, and is part of the order *Nidovirales* [14]. PRRSV is further classified into two species, *Betaarterivirus suid 1* (PRRSV-1 or EU type) and *Betaarterivirus suid* *2* (PRRSV-2 or NA type), based on their antigenicity [15]. There were approximately 60% genomic similarities between PRRSV-1 and PRRSV-2. Within each genotype, variations in the nucleotide sequence occur of up to 20% [16]. Based on the phylogenetic analysis of ORF5, PRRSV-1 was classified into four subtypes: subtype 1 (Global), subtype 1 (Russia), subtype 2, and subtype 3. In addition, subtype 1 (Global) is further divided into 12 clades labeled A-L. PRRSV-2 comprises nine lineages, specifically lineages 1 through 9 [17, 18].

Since the first report of PRRSV in 1989 [19], the virus has been circulating among Thai swine for the past three decades. PRRSV remains a primary RNA virus that significantly affects Thai pig livestock, leading to high mortality rates and substantial production losses in the swine industry across Thailand. Nationwide swine herds in Thailand have been identified as hosting both PRRSV-1 and PRRSV-2 based on epidemiological studies [9, 20, 21]. According to studies on the genetic diversity and epidemiology of PRRSV in Thailand, lineages 1, 5, and 8 are associated with PRRSV-2, whereas subtype 1 (Global) clades A, D, and H have been linked to PRRSV-1 [9]. Concurrent infections and mixed infections of PRRSV-1 and PRRSV-2 have been detected in swine farms in Thailand [9, 20].

Despite extensive research on the two PRRSV variants, most notably PRRSV-2, the spread and origins of PRRSV-1 on pig farms have not yet been determined. Understanding and comprehending the distribution of PRRSV-1, specifically the origins of disease transmission, can be beneficial for disease prevention, biosecurity protocols, and the enhancement of vaccine efficacy, as well as strategies for PRRSV control. Therefore, the objectives of this study were to determine the origin of the infected pigs that caused disease transmission within the farms and to investigate the distribution of PRRSV-1 in the pig production chain across three pig-fattening farms. The findings of this study can be used as recommendations for improvements and more effective planning to reduce the spread of PRRSV and manage the disease in swine farms.

## Materials and Methods

### Ethical approval

The Institutional Animal Care and Use Committee of Mahanakorn University of Technology approved the study (approval no. ACUC-MUT-010), confirming that it adheres to the standards set by the Ethical Review Board of the National Research Council of Thailand for the care and use of animals in scientific research.

### Study period and location

This retrospective study used specimens gathered over 17 months across four farms in three provinces of Thailand. Nakhon Pathom province is located in Central Thailand, while Chonburi and Sa Kaeo are located in Eastern Thailand. The study area was selected according to the geographic patterns of pig production systems, and these provinces represent the intensive pig sector.

### Pig farms and production systems

**Figure-1:**
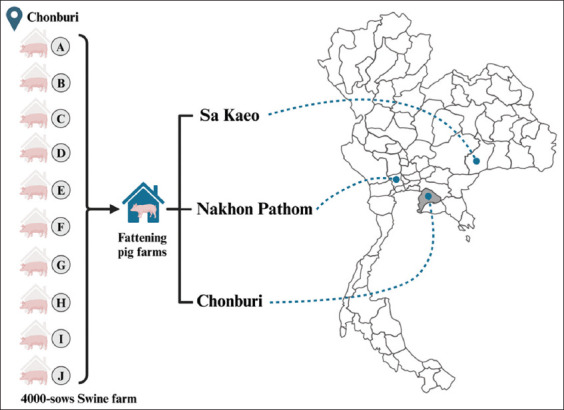
The pig farms in this study are situated in Central Thailand (Nakhon Pathom) and Eastern Thailand (Chonburi and Sa Keao). The breeding farm (grey area in the map), housing a total of 4,000 sows, is located in Chonburi province and comprises 10 pig houses. After being weaned at 4 weeks of age, the finishing pigs are transferred to pig-fattening farms in Chonburi, Sa Kaeo, and Nakhon Pathom provinces. Each of these farms has a capacity of 500–600 pigs. (Source: This image was created using BioRender scientific illustration software, and the map was constructed using Microsoft Excel in Microsoft Office LTSC Professional Plus 2021).

For this study, four farms were used: One breeding farm housing 4,000 sows and three pig-fattening farms, each with an average of 500–600 pigs. Gilts, sows, and finishing pigs were administered vaccinations with PRRSV-2-modified live virus. The breeding farm in the Chonburi province of Thailand comprises ten pig houses (A-J), each accommodating 400 sows. The buildings housed the gestation area for sows, as well as areas for pregnant sows and gilts awaiting mating and a farrowing house for piglets until they were weaned. At the age of 4 weeks, the finishing pigs were transported to pig-fattening farms in the provinces of Chonburi, Sa Keao, and Nakhon Pathom (Figure-1). Pigs at these three farms were cared for from 4 to 24 weeks of age using an “all-in-all-out” operational approach.

### Sample collection

A total of 696 samples were randomly collected and categorized into three groups: Group 1, group 2, and group 3. Group 1 comprised a random selection of 310 serum samples obtained from sows and gilts at breeding farms located in Chonburi province. The estimated prevalence of PRRS was approximately 1%, with a 95% confidence level. Group 2 included 343 serum samples collected at different ages; 4, 8, 12, 18, and 24 weeks – from fattening pig farms in a randomized manner. The disease prevalence within these farms was assessed as 30%, with a confidence level of 95%. Group 3 comprised an additional 43 additional samples that were procured beyond the anticipated sample volume. These included carcasses of finishing pigs exhibiting respiratory symptoms and placental samples from both healthy and unhealthy sows. A summary of the total samples used in this study included 310 samples from a breeding farm in Chonburi province, 90 samples from pig-fattening farms in the same province, 58 samples from a farm in Nakhon Pathom province, and 238 samples from a farm in Sa Kaeo province. In total, 696 samples were collected from all three groups (Table-1).

**Table-1:** The sample data used in the study.

Sampling	Samples	Number of samples/Provinces			Total

		Chonburi	Nakhon Pathom	Sa Kaeo	
Group 1	Serum samples of gilts and sows	310	-	-	310
Group 2	Serum samples of fattening pigs				343
	4 weeks	20	15	60	95
	8 weeks	12	12	58	82
	12 weeks	10	11	50	71
	18 weeks	10	10	30	50
	24 weeks	10	10	25	45
Group 3	Other samples				43
	Placenta	20	-	-	20
	Carcasses of finishing pigs	8	-	15	23
Total		400	58	238	696

### RNA extraction and cDNA synthesis

Total RNA was extracted from serum samples and tissue homogenates using a viral RNA extraction kit (Geneaid, Taiwan) per the manufacturer’s instructions. The isolated RNA was subsequently reverse transcribed using oligo(dT) primers and Moloney Murine Leukemia Virus (M-MuLV) Reverse Transcriptase (Vivantis Technologies, Malaysia) following the manufacturer’s protocol. The cDNA synthesis was carried out at 42°C for 60 min and then at 85°C for 5 min. This was performed in the presence of a reaction mixture that comprised 8 μL of the RNA, 1 μL oligo(dT), 1 μL dNTP, 7 μL RNase-free H_2_O, 2 μL 10× Buffer M-MuLV, and 1 μL M-MuLV Reverse Transcriptase.

### The reverse transcription polymerase chain reaction (RT-PCR)

All samples were initially screened for PRRSV using ORF7-specific primers (Forward primer: GCCCCTGCCCAYCACG; Reverse primer: TCGCCCTAATTGAATAGGTGA) [22]. Subsequently, the positive samples were classified as PRRSV-1 using ORF5-specific primers (Forward primer: CAATGAGGTGGGCIACAACC; Reverse primer: TATGTIATGCTAAAGGCTAGCAC), with a PCR fragment size of 719 bp [9]. The cDNA served as the template in the PCR with a final volume of 100 μL. This process included 10 pmol of each of the PRRSV-specific primers mentioned earlier, 10× Taq polymerase buffer, 10 mM dNTP, 25 mM MgCl_2_, and 2.5 units of Platinum^®^ *Taq* DNA polymerase (Invitrogen, USA). For ORF7, PCR was conducted under the following conditions in a thermal cycle: Initial denaturation at 94°C for 2 min; 35 cycles of denaturation at 94°C for 30 s, annealing at 58°C for 40 s, and elongation at 72°C for 40 s; and a final cycle at 72°C for 7 min. For ORF5, the PCR cycles consisted of primary denaturation at 94°C for 3 min, followed by 40 cycles of denaturation at 94°C for 30 s, annealing at 56°C for 40 s, and elongation at 72°C for 1 min, with a final elongation at 72°C for 10 min. The amplified products were analyzed by electrophoresis in 1.2% agarose gel and visualized under ultraviolet light.

### Sequence analysis

The PCR products were purified using a Gel/PCR Fragments Extraction Kit (RBC Bioscience, Taiwan) according to the manufacturer’s instructions. The purified products were then sent to a commercial laboratory for sequencing with forward and reverse primers (Macrogen, Korea). Full-length ORF5 DNA sequences were assembled using the Segman application provided in the Lasergene Molecular Biology software (DNAStar, USA). After a thorough analysis of all ORF5 nucleotide sequences, PRRSV-1 sequences were selected to represent this study. This selection was based on the similarity of nucleotide sequences (% identity); sequences with >97% ORF5 nucleotide similarity were considered to belong to the same strain [23]. The 107 representative PRRSV-1 strains were classified into subtypes and clades by phylogenetic analysis using the maximum likelihood method with 1000 replicates of the comparison provided in MEGAX software (https://www.megasoftware.net/). The information on all PRRSV sequences used in this study is available as supplementary data.

To investigate genetic variation at the amino acid level, the nucleotide sequences of ORF5 were translated into GP5 amino acid. Multiple sequence alignments were then performed for comparison with PRRSV-1 reference sequences from GenBank. This analysis was performed using the ClustalW algorithm with the Lasergene Molecular Biology software (DNAStar, USA). The NetNGlyc 1.0 Server, https://services.healthtech.dtu.dk/services/NetNGlyc-1.0/, was employed to identify the putative N-linked glycosylation sites (NGS).

## Results

### Detection of PRRSV-1 infection

Of the 696 samples collected from four pig farms, 28 were positive for PRRSV-1 using ORF5 RT-PCR, and only pig-fattening farms were found to be PRRSV-1-positive. Following genetic analysis, which removed several sequences with significant similarity and very close relatedness, 13 positive samples were selected to serve as representative PRRSV-1 samples for the study. These PRRSV-1 strains were all from finishing pigs at Nakhon Pathom (n = 1), Chonburi (n = 4), and Sa Kaeo (n = 8) farms. The PRRSV-1 sequences are described in Table-2.

**Table-2:** Information regarding 13 representative PRRSV-1 collected from finishing pigs in three provinces of Thailand.

Province	Animals	PRRSV strains	Date of sampling	Age (week)	Type of sample
Nakhon Pathom	Finishing pig	EU/TH/NPT011/2014	October 23, 2014	4	Serum
Chonburi	Finishing pig	EU/TH/CBI005/2015	January 10, 2015	13	Lung
Chonburi	Finishing pig	EU/TH/CBI030/2015	May 06, 2015	6	Serum
Chonburi	Finishing pig	EU/TH/CBI033/2015	May 06, 2015	6	Serum
Chonburi	Finishing pig	EU/TH/CBI034/2015	May 06, 2015	6	Serum
Sa Kaeo	Finishing pig	EU/TH/SKW012/2015	March 05, 2015	11	Serum
Sa Kaeo	Finishing pig	EU/TH/SKW015/2015	March 05, 2015	11	Serum
Sa Kaeo	Finishing pig	EU/TH/SKW051/2015	July 15, 2015	10	Lung
Sa Kaeo	Finishing pig	EU/TH/SKW052/2015	July 15, 2015	8	Lung
Sa Kaeo	Finishing pig	EU/TH/SKW165/2015	July 21, 2015	6	Serum
Sa Kaeo	Finishing pig	EU/TH/SKW170/2015	July 21, 2015	6	Serum
Sa Kaeo	Finishing pig	EU/TH/SKW179/2015	July 21, 2015	9	Serum
Sa Kaeo	Finishing pig	EU/TH/SKW029/2016	January 18, 2016	9	Serum

PRRSV=Porcine reproductive and respiratory syndrome virus-1

### Analysis of ORF5 nucleotide sequences

**Figure-2:**
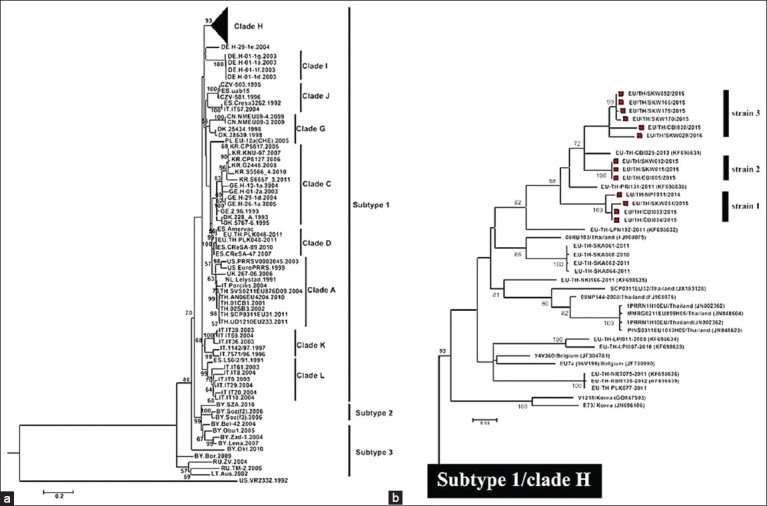
Phylogenetic tree based on the complete ORF5 gene, demonstrating genetic classification between Thai PRRSV-1 strains and reference PRRSV-1 strains. (a) A total of 107 ORF5 sequences, including 13 from Thai PRRSV-1 strains in this study, 93 PRRSV-1 reference sequences from the GenBank database, and the PRRSV-2 strain VR2332 (U87392) as the outgroup, were used in the phylogenetic analysis. The PRRSVs grouped in subtype 1/clade H are shown with black triangles. (b) PRRSV-1 strains in subtype 1/clade H include 13 Thai PRRSV-1 strains from this study, divided into three major strains, 20 Thai PRRSV-1 strains from previous studies, and four PRRSV-1 strains from Belgium and Korea. Thai PRRSV-1 strains from this study are labeled with red squares. The bootstrap values greater than 50% are shown on the branches. ORF=Open reading frames, PRRSV-1=Porcine reproductive and respiratory syndrome virus-1.

Nucleotide homology analysis was conducted to identify similarities among the complete ORF5 sequences of the 13 PRRSV-1 strains. The analysis revealed a notable range of percentage identity exhibited within the PRRSV-ORF5 sequences of these strains, ranging from 91.7% to 100%. Notably, the PRRSV-1 strains demonstrated a close relationship with EU/TH/CBI025/2013 (KF698631), as our analysis revealed a percentage identity in the range of 94%–96%. The reference strain EU/TH/CBI025/2013 was initially identified by our research group in 2013 in Chonburi province, Thailand, and it was taxonomically classified as PRRSV-1 subtype 1/clade H [9]. Furthermore, our findings indicated that all 13 PRRSV-1 strains were grouped within PRRSV-1 subtype 1/clade H, which is consistent with the characteristics of the reference strain as determined using the construction of a phylogenetic tree (Figure-2).

### Distribution patterns of PRRSV-1 in three swine farms

The distribution patterns of PRRSV-1 were further investigated within the swine production chain, extending from the breeding farm comprising ten pig houses (A-J) to three pig-fattening farms. The distribution patterns of PRRSV-1 were classified into three groups based on the pig houses in the breeding farm, which served as the initial housing for the pigs before they were moved to the three pig-fattening farms. Group 1 involved the transfer of piglets from breeding farm house A in Chonburi to pig-finishing farms in Nakhon Pathom, Chonburi, and Sa Kaeo. The EU/TH/NPT011/2014 strain was isolated from 4-week-old piglets in Nakhon Pathom on October 23, 2014. The EU/TH/CBI033/2015 and EU/TH/CBI034/2015 strains were isolated from 6-week-old piglets in Chonburi on May 6, 2015. In addition, the EU/TH/SKW051/2015 strain was isolated from 10-week-old piglets in Sa Kaeo on July 15, 2015. All four strains exhibited >97% identity, indicating that they were the same strain (Table-3). Group 2 involved the transfer of piglets from Chonburi’s breeding farm (pig house F) to pig-finishing farms in Chonburi and Sa Kaeo. On January 10, 2015, the EU/TH/CBI005/2015 strain was identified in 13-week-old finishing pigs in Chonburi. Subsequently, on March 5, 2015, the EU/TH/SKW012/2015 and EU/TH/SKW015/2015 strains were isolated from 11-week-old finishing pigs in Sa Kaeo. The sequence identities of all four strains exceeded 97%, indicating their classification as the same strain (Table-3). Group 3 transported piglets from Chonburi’s breeding farm (pig house H) to pig-finishing farms in both Chonburi and Sa Kaeo. On May 6, 2015, the EU/TH/CBI030/2015 strain was identified in 6-week-old finishing pigs in Chonburi. Subsequently, on July 15, 2015, the EU/TH/SKW052/2015 strain was identified in 8-week-old finishing pigs in Sa Kaeo. Furthermore, the EU/TH/SKW165/2015 strain was discovered in 6-week-old finishing pigs in Sa Kaeo on July 21, 2015. In Sa Kaeo, EU/TH/SKW170/2015 was found in 6-week-old finishing pigs, whereas the EU/TH/SKW179/2015 strain was found in 9-week-old finishing pigs. Subsequently, the EU/TH/SKW029/2016 strain was discovered in 9-week-old finishing pigs in Sa Kaeo on January 18, 2016. The sequence identities of all six strains exceeded 97%, indicating that they were the same strain (Table-3).

**Table-3:** Distribution patterns of PRRSV-1 from breeding farm to three pig-fattening farms.

Major strain	Minor strain	House/Province	Sampling date	Breeding farm	

				Province	House
1	EU/TH/NPT011/2014	Nakhon Pathom	October 23, 2014	Chonburi	A
	EU/TH/CBI033/2015	Chonburi	May 06, 2015	Chonburi	A
	EU/TH/CBI034/2015	Chonburi	May 06, 2015	Chonburi	A
	EU/TH/SKW051/2015	Sa Kaeo	July 15, 2015	Chonburi	A
2	EU/TH/CBI005/2015	Chonburi	January 10, 2015	Chonburi	F
	EU/TH/SKW012/2015	Sa Kaeo	March 05, 2015	Chonburi	F
	EU/TH/SKW015/2015	Sa Kaeo	March 05, 2015	Chonburi	F
3	EU/TH/CBI030/2015	Chonburi	May 06, 2515	Chonburi	H
	EU/TH/SKW052/2015	Sa Kaeo	July 15, 2515	Chonburi	H
	EU/TH/SKW165/2015	Sa Kaeo	July 21, 2015	Chonburi	H
	EU/TH/SKW170/2015	Sa Kaeo	July 21, 2015	Chonburi	H
	EU/TH/SKW179/2015	Sa Kaeo	July 21, 2015	Chonburi	H
	EU/TH/SKW029/2016	Sa Kaeo	January 18, 2016	Chonburi	H

PRRSV=Porcine reproductive and respiratory syndrome virus-1

Subsequently, all 13 representative strains of PRRSV-1 were eventually grouped into major strains 1, 2, and 3, aligning with the distribution patterns of the PRRSV-1 virus originating from various pig houses on the same breeding farm to pig-finishing farms (Table-3). In detail, major strain 1 comprises EU/TH/NPT011/2014, EU/TH/CBI033/2015, EU/TH/CBI034/2015, and EU/TH/SKW051/2015. The distribution patterns of PRRSV-1 in these strains originated from pig house A of a breeding farm in Chonburi and were extended to pig-finishing farms in Nakhon Pathom, Chonburi, and Sa Kaeo provinces of Thailand. Major strain 2 consisted of EU/TH/CBI005/2015, EU/TH/SKW012/2015, and EU/TH/SKW015/2015, with distribution patterns from pig house F of the breeding farm in Chonburi to pig-finishing farms in Chonburi and Sa Kaeo provinces of Thailand. Major strain 3 included EU/TH/CBI030/2015, EU/TH/SKW052/2015, EU/TH/SKW165/2015, EU/TH/SKW170/2015, EU/TH/SKW179/2015, and EU/TH/SKW029/2016. The distribution patterns of PRRSV-1 in these strains extend from pig house H of the breeding farm in Chonburi to pig-finishing farms in Chonburi and the Sa Kaeo provinces of Thailand (Table-3).

### Amino acid analysis of GP5

To explore genetic variation at the amino acid level, the DNA sequences of ORF5 were translated into GP5 amino acid sequences. The amino acid sequences of 13 representative PRRSV-1 strains (major strains 1–3) and 12 PRRSV-1 reference strains were meticulously analyzed. These reference strains were isolated during the PRRSV-1 outbreak in Thailand between 2008 and 2015. The list of 12 PRRSV-1 reference strains [9] comprises two strains previously identified in Chonburi province (EU/TH/CBI025/2013 and EU/TH/CBI004/2015) (major strains 4) along with PRRSV-1 outbreaks in other provinces (n = 9) in Thailand. These strains included EU/TH/PRI131/2011, EU/TH/LPI011/2008, EU/TH/LPI012/2008, EU/TH/NKI166/2011, EU/TH/NRT075/2011, EU/TH/PLK048/2011, EU/TH/SKA060/2010, EU/TH/SKA061/2010, and EU/TH/SKA06/2010 (major strains 5). Additionally, the Lelystad strain (M96262), which served as the PRRSV-1 prototype, was included in the analysis.

**Figure-3:**
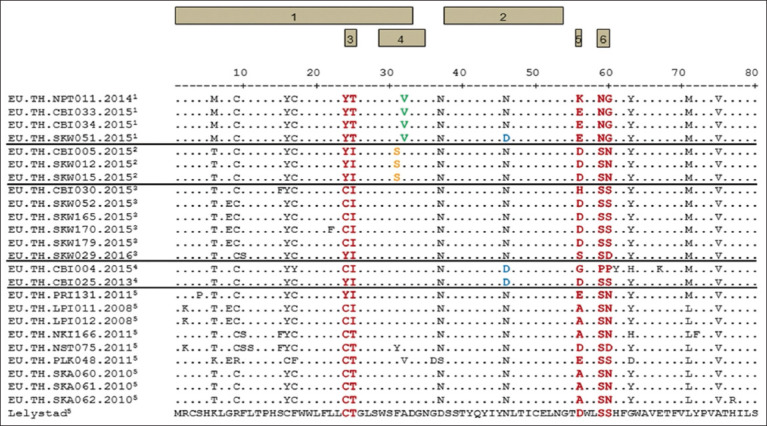
The amino acid alignment of GP5 was conducted among 13 representative PRRSV-1 strains, 11 PRRSV-1 reference strains, and the Lelystad strain (PRRSV-1 prototype). The numbered categories include: (1) PRRSV-1 major strain 1, (2) PRRSV-1 major strain 2, (3) PRRSV-1 major strain 3, (4) PRRSV-1 strain isolated in Chonburi province from our previous study, and (5) PRRSV-1 strain isolated in a different province of Thailand. The boxes labeled 1–6 above the amino acid sequence correspond to: (1) Signal peptide (amino acid positions 1–33), (2) Neutralizing epitope B (amino acid positions 38–54), (3) Hypervariable region 1 (HVR1), (4) Non-neutralizing epitope A (amino acid positions 29–35) in the Lelystad strain, (5) Hypervariable region 2 (HVR2), and (6) Hypervariable region 2 (HVR2). PRRSV-1=Porcine reproductive and respiratory syndrome virus-1.

The GP5 of PRRSV-1 is divided into five domains, progressing from amino to carboxy termini: signal peptide (residues 1–33), ectodomain, two transmembrane domains, and endodomain. Within the ectodomain, GP5 encompasses two B cell epitopes: Decoy epitope or non-neutralizing epitope A (residues 29–35) [24] and neutralizing epitope B (residues 38–54) [25]. Multiple alignments revealed that the N-terminal regions of GP5 in all 25 PRRSV-1 strains exhibit high variability, while the C-terminal regions were more conserved. In this study, all 25 PRRSV-1 strains included hypervariable regions located at amino acid positions 24–25, 56, and 59–60 in GP5 amino acid sequences (Figure-3). All 25 PRRSV-1 strains also share a conserved region on neutralizing epitope B at amino acid positions 38-54 (38-SSTYQYIYNLTICELNG-54) [25]. Notably, amino acid position 46 undergoes a change from Asparagine (N) to Aspartic acid (D) in EU/TH/CBI004/2015, EU/TH/CBI025/2013 (major strains 4), and EU/TH/SKW051/2015 (major strain 1) (Figure-3). As described previously, non-neutralizing epitope A is a conserved motif containing amino acid sequence 29WSFADGN35 in the Lelystad strain [24]. Specifically, all four PRRSV-1 strains of major strain 1 exhibited amino acid variation at position 32 from Alanine (A) to Valine (V). In contrast, all three PRRSV-1 strains of major strain 2 exhibited amino acid variation at position 31 from Phenylalanine (F) to Serine (S) (Figure-3).

### Analysis of potential NGSs on GP5

Our study further analyzed N-linked glycosylation patterns within GP5 among 13 representative PRRSV-1 strains, 11 PRRSV-1 reference strains, and the Lelystad strain (PRRSV-1 prototype). The results demonstrated that N-glycan was added to the GP5 protein at amino acid positions 37, 46, and 53, which are located on neutralizing epitope B (amino acid positions 38–54). Among the 25 PRRSV-1 sequences, three patterns of N-linked glycosylation of the GP5 protein were identified (Table-4). Pattern 1 had three glycosylation sites; N-linked glycosylation was added at amino acid positions 37, 46, and 53 in strains with the amino acid sequence NSSTYQYIYNLTICELN at amino acid positions 37–53. This pattern was primarily observed in 21 PRRSV-1 strains in our study. Pattern 2 involved N-linked glycosylation additions at positions 37 and 53, and the amino acid sequences from positions 37 to 53 were designated NSSTYQYIYDLTICELN. This pattern was observed in EU/TH/CBI004/2015, EU/TH/CBI025/2013 (major strain 4) and EU/TH/SKW051/2015 (major strain 1). Pattern 3 showed N-linked glycosylation added at amino acid positions 46 and 53, which were found in strains with amino acid sequences from positions 37 to 53 as DSSTYQYIYNLTICELN of the Lelystad strain (Table-4).

**Table-4:** The N-linked glycosylation patterns found in GP5 of PRRSV-1 on positions 37–53.

Glycosylation pattern	Amino acid site																	Sequence number

	37	38	39	40	41	42	43	44	45	46	47	48	49	50	51	52	53	
1	N	S	S	T	Y	Q	Y	I	Y	N	L	T	I	C	E	L	N	21
2	N	S	S	T	Y	Q	Y	I	Y	D	L	T	I	C	E	L	N	3
3	D	S	S	T	Y	Q	Y	I	Y	N	L	T	I	C	E	L	N	1

The red letters indicate N-linked glycosylation sites. PRRSV-1=Porcine reproductive and respiratory syndrome virus-1

## Discussion

The PRRSV is undoubtedly the most well-known swine virus that has significantly impacted the Thai economy in the past 30 years. Unfortunately, efforts to eliminate PRRSV from the Thai swine population have been unsuccessful. One critical challenge is the high genetic diversity of PRRSV, which leads to the rapid emergence of new strains that diminish the efficacy of vaccines. In addition, information regarding the spread of PRRSV among swine herds in Thailand remains unclear. Gaining further insight into PRRSV’s distribution patterns throughout the swine production chain is necessary to control the disease and lessen its spread on the farm. To address this issue, the present study examined the distribution of PRRSV-1 across three pig-fattening farms in the pig production chain. This was performed by tracking pig movements from the breeding farm, which comprises pig houses A to J, to the three pig-fattening farms. PRRSV-1 strains were identified, and genetic characterization was performed.

Several epidemiological studies on PRRSV have revealed the co-circulation of both PRRSV-1 and PRRSV-2 in the Thai swine population [9, 20, 26, 27]. However, PRRSV-2 remains a primary concern among Thai pigs. As a result, veterinarians and related parties prioritize epidemiological studies exclusively on PRRSV-2, whereas PRRSV-1 remains less understood. To determine the distribution of PRRSV-1 among swine herds, we used RT-PCR to detect PRRSV-1 in 696 samples obtained from gilt, sow, and fattening pigs. The results revealed a positive rate of approximately 4.02% (28/696), consistent with earlier reports on the prevalence of PRRSV-1 in Thailand. One study documented a prevalence of 4.60% in Thailand during 2010 [27], while another reported varying prevalence ranging from 0.35% to 4.60% from 2008 to 2013 [9]. These findings indicate that PRRSV-1 persists as an endemic disease in the Thai swine population, contributing to sporadic outbreaks. Our investigation identified three primary PRRSV-1 subtype 1/clade H strains. Major strain 1 was widely distributed across pig-fattening farms in Nakhon Pathom, Chonburi, and Sa Kaeo. In contrast, strains from major strains 2 and 3 were predominantly found on Chonburi and Sa Kaeo farms. Notably, swine from Chonburi and Sa Kaeo exhibited a higher prevalence of diverse PRRSV-1 strains within their population, potentially leading to the emergence of novel strains. Thus, annually monitoring the emergence of new PRRSV strains in this particular swine herd is crucial.

GP5, an envelope protein subject to glycosylation, exhibits potent immunogenicity that induces the generation of neutralizing antibodies in pigs. This protein is critically important in diverse aspects of PRRSV, including diagnosis, phylogenetic analyses, vaccine development, and management and prevention of the virus [28]. In this study, genetic analysis of ORF5 revealed that all PRRSV-1 strains in pig-fattening farms were PRRSV-1 subtype 1/clade H. These strains closely resemble the EU/TH/CBI025/2013 (KF698631) strain from the Chonburi breeding farm [9], potentially serving as the prototype of PRRSV-1 in this pig production system. To deepen our understanding of the genetic diversity and evolution of these PRRSV-1 strains, we analyzed the antigenic determinants of GP5, comparing them with sequences from previous Thai PRRSV-1 strains. These findings align with established theories regarding the structural and antigenic features of GP5 in PRRSV-1 [25, 29]. However, major strains 1 and 2 of PRRSV-1 displayed unique features at amino acid positions 32 and 31, respectively. Our results are consistent with previous findings showing a relatively conserved pattern within the neutralizing epitope of PRRSV-1 [30–32]. In general, N-linked glycosylation plays a crucial role in proper protein folding and contributes to the biological activity and antigenicity of viral proteins. In PRRSV, N-linked glycans mask antigenic epitopes, preventing their recognition by neutralizing antibodies and enabling viral escape during persistent infection [33, 34]. Through N-linked glycosylation analysis, it was observed that almost all PRRSV-1 strains (major strains 1–3), except EU/TH/SKW051/2015 (major strain 1), possess three conserved NGSs at amino acid positions N37, N46, and N53 (glycosylation pattern 1), similar to previous Thai PRRSV-1 subtype 1/clade H [9]. Taken together, these findings suggest a relatively stable mutation rate among PRRSV-1 subtype 1/clade H strains in this pig farming system.

Conducting research on the distribution of PRRSV is essential for the implementation of eradication programs and the effective management of swine health [35, 36], which forms the core focus of this study. To understand the patterns of PRRSV-1 distribution in swine farms, we collected 13 representative PRRSV-1 strains. Our examination focused on tracking viral dissemination among isolates obtained from three pig-fattening farms and one breeding farm. The results revealed that PRRSV-1 strains within major strain 1 circulated among three pig-fattening farms, suggesting potential virus transmission originating from pigs housed in pig house A at the breeding farm. Regarding PRRSV-1 strains within major strains 2 and 3 circulating in farms located in Chonburi and Sa Kaeo, probable virus transmission occurred from pigs housed in pig houses F and H at the breeding farm, respectively. However, despite the data on PRRSV-1 distribution indicating the transfer of viruses from breeding farms to pig-finishing farms, this investigation did not detect any infection in gilts and sows. This discrepancy could be due to the potential latent infection of these pigs with PRRSV-1 during the investigation, in which the virus might have been undetectable at certain points. Other studies have yielded similar results, reinforcing the conclusions drawn in this regard [37, 38]. Nonetheless, positive samples from gilts and sows can be discovered if the study duration or sample collection is extended continuously.

Understanding PRRSV transmission reveals that infection spreads within pig herds through various risk factors involving both direct and indirect contact and manifests horizontally and vertically [39]. Horizontal transmission occurs when sick or infected pigs transmit PRRSV to healthy pigs through contact with bodily secretions such as blood, semen, saliva, feces, urine, mucus, milk, and colostrum. In contrast, vertical transmission occurs when gestating sows carrying PRRSV toward the end of gestation pass the virus through their milk to piglets [40]. Vertical transmission plays a crucial role in the persistence of PRRSV infection, even with vaccination in swine farms. This transmission mode can persist even if sows have acquired partial immunity through repeated vaccinations or contact with the wild-type virus [41]. Therefore, the challenge of controlling and preventing the spread of PRRSV-1 in pig-fattening farms is heightened by the persistent vertical transmission from sows in the middle to the end of gestation to piglets. This collective evidence strongly supports our conclusion that PRRSV-1 is transmitted from the breeding farm to the three pig-fattening farms.

Another perspective to consider is the possibility that PRRSV-1 strains present in pig-finishing farms may not have originated from the breeding farm. The discovery of PRRSV-1 in finishing pig farms, even when testing for PRRSV-1 in breeding pigs yields negative results, suggests that the biosecurity measures, whether external or internal, implemented on pig-finishing farms, may not be sufficiently stringent. Existing risk factors influences the infection dynamics. Despite negative sowing test results, this does not ensure the complete absence of the virus in the transported piglets. During the transportation of piglets to various finishing pig farms in different provinces, one significant risk factor that could impact piglet infection is the driver and the vehicle used for transporting pigs from one farm to another. Despite the process of cleaning and disinfecting the vehicle before unloading the pigs onto the farm, there are still several critical points on the vehicle that may not be reached or are difficult to clean, such as corners or small gaps. Furthermore, the driver is an additional potential risk factor for introducing the virus into the pig farm [42]. During transportation, the risk of piglet infection escalates when traversing PRRS-endemic areas, consistent with the negative findings in sows. Reports from the past have extensively documented the spread of PRRS in Thailand, specifically citing three provinces: Chonburi, Sa Kaeo, and Nakhon Pathom [9]. A previous study delved into the airborne capabilities of PRRSV, revealing its potential to travel distances of up to 4.7 km [43]. Furthermore, earlier research underscores the vulnerability of the Thai pig movement network to infectious diseases. Once these pathogens breach the system, they can rapidly disseminate throughout the country [44]. This cumulative information emphasizes the intricate dynamics of PRRS transmission and highlights the importance of vigilant measures during pig transportation in areas prone to the virus.

In the context of this study, finishing pig farms are considered medium-sized, consisting of several pig houses. The “all-in/all-out” system involves moving pigs into or out of the house together in batches, but this does not necessarily mean that all finishing pigs in every house on the farm are moved simultaneously. Consequently, new batches of pigs brought into a farm may be exposed to the virus circulating inside the farm. To address the risk of a PRRS outbreak, the use of a PRRSV-1 vaccine for pigs is considered a preventive measure because finishing pigs infected with PRRSV have a significantly higher mortality rate than sows [45]. Moreover, vaccines can stimulate immunity in pigs, facilitating disease prevention and reducing the spread of PRRSV to other pig farms [46]. However, further investigation is needed to identify the specific risks of PRRSV-1 infection in finishing pig farms.

## Conclusion

The investigation reveals how PRRSV-1 spreads through the swine production chain, connecting breeding farms to infections at fattening farms. The PRRSV-1 strain categorization corresponds to their dissemination from specific breeding farm pig houses to diverse pig-fattening farms. The theory of localized transmission from breeding to fattening farms is supported by this finding. Understanding these transmission pathways highlights the significance of controlling PRRSV-1 in breeding farms to avert its dissemination elsewhere, which would likely lessen economic losses and enhance swine health.

Our study sheds light on PRRSV-1 distribution in Thai swine herds, but it has certain limitations. Our sample size may not accurately represent the entire swine population in Thailand. Examining just three swine farms in Thailand might not reveal the overall epidemiological patterns among all swine farms. The limited scope of the study may impact the precise representation of PRRSV-1 in Thai swine populations. Our detection methods, though robust, may not identify all latent or low-level infections. The absence of detectable PRRSV-1 in gilts and sows could be due to the presence of the virus in undetectably low levels or in a latent state during sampling. The retrospective study design impedes the determination of causal relationships regarding the specific transmission routes of the virus. Continuous monitoring in prospective studies would yield more conclusive data on transmission dynamics.

Increasing the sample size and geographical diversity for PRRSV-1 studies in Thailand would enhance our understanding of its distribution and transmission patterns. Longitudinal studies tracking PRRSV-1 over extended periods can identify emerging strains, elucidate the virus’s evolution, and investigate the impact of environmental factors and human activities on its dissemination. Assessing the effectiveness of biosecurity measures and vaccination programs can be enhanced through this approach, crucial for improving vaccines and control strategies.

## Data Availability

The supplementary data can be available from the corresponding author on a reasonable request.

## Authors’ Contributions

TJ, TB, and PL: Conceptualization. TJ, WS, TB, and PL: Methodology. TJ and WS: Software. TJ, WS, and PL: Validation. TJ and WS: Formal analysis and visualization. TJ, WS, TB, WP, NC, TT, and PM: Investigation and Writing-original draft preparation. TJ, WS, and TB: Data curation. TJ, TB, WP, and NC: Writing-review and editing. PL: Supervision and project administration. All authors have read, reviewed, and approved the final manuscript.
